# Editorial: A Compendium of Recent Research on Stem Cell-Based Therapy for Covid-19

**DOI:** 10.3389/fcell.2021.813384

**Published:** 2021-12-14

**Authors:** Abdelkrim Hmadcha, Bernat Soria, Robert C. Zhao, Tarik Smani, Israel Valverde

**Affiliations:** ^1^ Department of Biotechnology, University of Alicante, Alicante, Spain; ^2^ University of Pablo de Olavide, Seville, Spain; ^3^ Spanish Biomedical Research Centre in Diabetes and Associated Metabolic Disorders (CIBERDEM), Madrid, Spain; ^4^ Department of Physiology, Institute of Bioengineering-ISABIAL, University Miguel Hernández School of Medicine, Alicante, Spain; ^5^ Institute of Basic Medical Sciences, Chinese Academy of Medical Sciences, School of Basic Medicine, Peking Union Medical College, Beijing, China; ^6^ School of Life Sciences, Shanghai University, Shanghai, China; ^7^ International Society on Aging and Disease, Bryan, TX, United States; ^8^ Cardiovascular Pathophysiology, Institute of Biomedicine of Seville, University Hospital of Virgen del Rocío, University of Seville, CSIC, Seville, Spain; ^9^ Department of Medical Physiology and Biophysics, University of Seville, Seville, Spain; ^10^ Pediatric Cardiology Unit, Virgen del Rocio University Hospital, Seville, Spain; ^11^ Institute of Biomedicine of Seville, University Hospital of Virgen del Rocío, University of Seville, CSIC, Seville, Spain

**Keywords:** advanced therapies, clinical trials, cell therapy, mesenchymal stromal cells, CAR T cells, COVID-19, cytokine storm, immunomodulaion

## Introduction

We have been dealing with an unprecedented global health crisis for nearly 2 years now. This started after an outbreak of atypical pneumonia of unknown etiology that was described in late December 2019 in China’s Wuhan Province, the etiologic agent causing this pneumonia episode was identified as a novel coronavirus named “Severe Acute Respiratory Syndrome CoronaVirus-2” (SARS-CoV-2)" (Coronaviridae Study Group of the International Committee on Taxonomy of Viruses, 2020; Wu et al., 2020) and the disease was designated COronaVIrus Disease-2019 (COVID-19). The rapid expansion of COVID-19 cases in number and geographic distribution led the World Health Organization (WHO) to declare a global health emergency. The control of the disease was challenged by the lack of antiviral treatment and vaccines, by asymptomatic carriers and the rapid increase in infections worldwide; COVID-19 was officially classified and declared by WHO as a pandemic on March 11, 2020 (WHO, 2020).

COVID-19 affects people differently, the majority of infected individuals develop mild to moderate disease and recover without hospitalization, but a subgroup of patients progresses to severe disease, with a high mortality rate and limited treatment options. However, the clinical features of COVID-19 vary from asymptomatic forms to conditions involving multi-organ and systemic manifestations in terms of septic shock and multiple organ dysfunction syndrome (MODS). Common primary pathologic features of critical COVID-19 overlap with acute lung injury (ALI) and acute respiratory distress syndrome (ARDS). While most infected individuals are usually asymptomatic or have mild symptoms, about 15% are affected by ARDS, of which 5% progress to multiple organ dysfunction syndrome or failure (Hui and Zumla, 2019).

COVID-19 involves direct attacks by SARS-CoV-2 on cells and secondary attacks on the body following activation of the immune system. Consequently, both the virus and the immune response can cause damage to the body, leading to common complications or secondary infection. The spike protein (S-protein) of SARS-CoV-2 binds to its functional receptor angiotensin-converting enzyme 2 (ACE2), triggering endocytosis of virus particles to infect target cells (Li et al., 2003; Tay et al., 2020). Therefore, potential therapeutic candidates for studying the mechanisms of SARS-CoV-2 infection include the ACE2 receptor (Zhang et al., 2020).

Given that the exact pathogenesis of SARS-CoV-2 and the dynamics of the disease are not yet fully understood, nor are specific antiviral options available, makes the treatment of patients with COVID-19 still a challenge (Tay et al., 2020); therefore, the available treatment options are limited. Several antiviral drugs (Grein et al., 2020), corticosteroids (Wang et al., 2020; Rasheedi et al., 2021), convalescent plasma (Shen et al., 2020; Verma et al., 2020) and neutralizing monoclonal antibodies (Shanmugaraj et al., 2020) have been tested and have undergone different phases of clinical trials, but none have been approved for COVID-19. Another alarming development is the recurrence of infection in recovered and even vaccinated individuals, which challenges the efficacy of current treatments (Lan et al., 2020). Under this situation, research has been carried out at an unprecedented speed to achieve a vaccine. Although several vaccines are now available and millions of people have received their full dose, the process of assessing safety, efficacy is still challenged, and a longer follow-up is still required (Kadkhoda, 2021; Olliaro et al., 2021; Yan et al., 2021). While the efficacy of vaccines has focused on the reduction of the number of symptomatic cases, the durability of acquired immunity following vaccination, the protection against reinfection by SARS-CoV-2 and its emerging variants remain to be proven and even improved (Townsend et al., 2021).

Thus, there is an urgent need for the development of feasible, safe and effective therapies. Therefore, growing experimental and clinical evidence suggests that advanced therapies could provide a potential therapeutic alternative for COVID-19 caused by SARS-CoV-2 and its emerging variants. In particular, cell therapy has been proven to significantly improve the sequelae related to COVID-19. In this regard, MSCs are among the most frequently used cell type for cell therapy, and considerable efforts have been made to introducing this advanced cell-based therapy into clinical practice. MSCs have been established as promising candidate sources for cell-based therapy their broad pharmacological effects and due to their contributions to tissue and organ homeostasis, repair and support by self-renewal and multi-differentiation, as well as by their anti-inflammatory, anti-proliferative, immunomodulatory, pro-angiogenic, pleiotropic, tropic and trophic properties. Various diseases have been successfully treated by MSCs in animal models and hundreds of clinical trials related to the potential benefits of MSCs are ongoing or have been successfully concluded so far. MSCs also secrete a myriad of soluble factors and vesicles altogether contribute to tissues and organs support, repair, homeostasis and functionality. The efficacy of MSCs and their secretory factors has been proven in successfully reducing inflammation, dampening immune responses and repairing lung damage in various pre-clinical and clinical models (Hmadcha et al., 2009). As described in the review by Ligotti et al., the key roles of both immunosenescence and immunopathology in the outcome of SARS-CoV-2 infection are supported by the beneficial results obtained with infusion of MSCs that act by restoring immune homeostasis and contributing to lung repair. In addition, the potential of MSC-based therapy as an option for severe or critically ill COVID-19 patients has been explored (Leng et al., 2020; Sánchez-Guijo et al., 2020). Several studies focus on regenerative, immunomodulatory, and anti-inflammatory properties of mesenchymal stromal cells (MSCs) to reduce the manifestation of cytokine storm and to restore ARDS and ALI, exhibiting an important option to be applied to critical COVID-19 patients; or on MSCs secretome to treat COVID-19 pneumonia (Li et al., 2020; Liang et al., 2020; Meng et al., 2020; Tang et al., 2020; Lanzoni et al., 2021). The contribution of Arjmand et al., summarizes the research on cytokine storm, one of the key causes of MODS as a hallmark of COVID-19 severity, highlights the benefit of stem cell-based therapies to attenuate cytokine release syndrome and suggests emerging advantages of MSCs secretome and extracellular vesicles (EVs) as treatment approaches for COVID-19. Besides, 2 interesting hypotheses and theories are formulated on the one hand, Nazerian et al., hypothesize that the use of chimeric 8P9R peptide and soluble ACE2 using exosome-liposome hybrids in the form of a two-step phase-dependent therapeutic strategy could inhibit the viral intracellular pathway and also inhibit progression in cytokine storm in a personalized manner. Given that this strategy is sensitive to the inflammatory status of individuals, it may improve the outcome of COVID-19 mortality. On the other hand, Babajani et al., provided new shreds of evidence about mitochondrial dysfunction and its effects on the immune response in COVID-19 and hypothesized that *in vivo* and *in vitro* experimental transferring healthy mitochondria by MSCs to damaged cells can provide a new therapeutic approach for COVID-19.

Within the cell therapy approach, other research includes the use of hematopoietic stem cells derived from umbilical cord blood, bone marrow, or mobilized peripheral blood, as well as immune chimeric antigen receptor T-cell (CAR-T cells) (Vardhana and Wolchok, 2020). Of note, an interesting immunology and virology original paper by Li et al., provided data that can further improve development of vaccines and new therapies against COVID-19. In this regard, authors reported a series of potential epitopes on SARS-CoV-2 spike glycoprotein which are recognized by CD4^+^ and CD8^+^ T cells from patients recovered from COVID-19 in China. The authors found that CD134^+^ and CD137^+^ T cells can react to an epitope outside the spike receptor domain (RBD), isolated T-cell receptor (TCR) sequences from the immunoreactive T cells and also inserted into Jurkat and CD4^+^ T cells to identify an epitope-specific TCR. Ferreras et al., for their part, characterized SARS-CoV-2-specific T-cell population within the CD45RA^−^ memory T cells (either CD4^+^ or CD8^+^) from blood of convalescent donors. In addition, the authors have demonstrated that these cells can be easily, effectively, and rapidly isolated following a donor selection strategy based on IFN-γ expression after exposure with SARS-CoV-2-specific peptides and HLA antigen expression, thereby obtaining clinical-grade CD45RA^−^ memory T cells, without requirements for GMP conditions, allowing the establishment of a biobank of SARS-CoV-2 specific memory T cells to be used to treat moderate to severe cases of COVID-19 patients.

The understanding of the mechanism of infection and pathogenesis also have great appeal and are still limited. In this regard; the use of human pluripotent stem cells, both embryonic stem cells (hESCs) and induced stem cells (hiPSCs), to generate tissue-specific human organoids (lung, intestinal, liver, vascular, heart, and kidney organoids) may provide a next-generation cellular model for investigating viral infection and drug screening (Yang et al., 2020). In this context, Larijani et al., reviewed the currently available iPSC-derived cells, iPSC-derived organoid models and animal models for studying the pathophysiology mechanisms of COVID-19-associated disorders and discussed the challenges and limitations that need to be overcome to optimize modelling approaches for the disease, and proposed potential therapeutic advances identified in experimental studies. Relatedly, in their review Luo et al., recapitulated findings on the application of hiPSC-derived cell models and organoids for COVID-19 to understand the action of SARS-COV-2 on human cells, underlining the importance of developing these models for long-term experiments not only to study the pathology of SARS-CoV-2 infection, respiratory failure and dysregulation of organs and systems, but also, to mimic the natural host-virus interaction, to clarify viral infection mechanisms and to elucidate the *in vivo* conditions of viral life cycles and drug screening.

We would like to conclude referring to the opinion by Zhao CR and others, pioneer in using MSCs to improve the outcome in patients with COVID-19 pneumonia (Leng et al., 2020). Wang et al., bring the debate about the clinical use of MSCs and MSCs-derived EVs for combating COVID-19, the risk of their uncontrolled commercial application to the forefront and the lack of any suggestions on regulations and guidelines for regulatory agencies to adopt new policies to prevent the sale of unproven MSC-based treatments in COVID patients.

Here we assembled a compendium of 9 manuscripts (2 original research, 4 reviews, 2 hypothesis and theory and 1 opinion) that cover the scope of this research topic, thus highlighting some of the recent advances and progresses of preclinical and clinical research and discussing some of the critical aspects related to the application of stem cell for COVID-19. Altogether, the ultimate goal of all these strategies is to achieve a safe and controlled therapy for COVID-19.
